# Draft genome sequence of perfluorinated acids C_7_–C_9_ degrading bacterium *Pseudomonas mosselii* 5(3), isolated from soil contaminated with pesticides

**DOI:** 10.1128/MRA.00839-23

**Published:** 2023-11-08

**Authors:** Sergey Chetverikov, Gaisar Hkudaigulov, Danil Sharipov, Sergey Starikov, Yanina Delegan

**Affiliations:** 1 Laboratory of Agrobiology, Ufa Institute of Biology, Subdivision of the Ufa Federal Research Centre of the Russian Academy of Sciences, Ufa, Russian Federation; 2 Federal Research Center-Pushchino Scientific Center for Biological Research of the Russian Academy of Sciences, Pushchino, Moscow, Russian Federation; University of Arizona, Tucson, Arizona, USA

**Keywords:** *Pseudomonas*, Perfluorinated carbone acids, draft genome, biodegradation, defluorination

## Abstract

The P*seudomonas mosselii* 5(3) strain is a potential degrader of persistent perfluorinated pollutants, particularly C_7_–C_9_ perfluorinated acids. The genome of the strain has been fully sequenced. It consists of a chromosome with a length of 5,676,241 base pairs and a G-C content of 64.38%.

## ANNOUNCEMENT

Halogenated pollutants top the list of persistent organic pollutants, including perfluorocarboxylic acids (PFCA), which are included in Annex B of the Stockholm Convention on Persistent Organic Pollutants (1–3). The bacterial transformation of these compounds is a key solution to the environmental contamination issue caused by these compounds. The *Pseudomonas mosselii* 5(3) strain was isolated from pesticide-contaminated arable soil (about 10 g) collected with a spoon from a 0–5 cm horizon in the Yanaulsky district, Republic of Bashkortostan, Russian Federation (56.113881°N, 54.766226°E). To do this, 1 g of soil was serially diluted to a sixfold amount and sown on Raymond’s agarized medium (4) with 100 ppm of C_8_ PFCA as the sole carbon source, followed by incubation for 7 days at 30°C. Unique colonies were restreaked on 100 ppm PFCA-agar plates, and 5(3) was isolated as a single colony. *P. mosselii* 5(3) grows aerobically in the presence of 0.01% C_7_–C_9_ PFCA as a sole carbon and energy source in Raymond’s medium (4) at 30°C with shaking. The growth is accompanied by the release of fluoride ions (up to 16 mg/L), which indicates the destruction process of PFCA. Strain 5(3) is deposited in the microorganism collection of the Ufa Institute of Biology as UIB-251.

For long-term storage, the strain was kept in glycerol (40%) stocks at −70°C. For short-term maintenance and biomass preparation, the strain was cultured on a lysogeny broth (LB) agar plate at 27°C.

To better understand the mechanisms of PFCA biodegradation, a draft genome sequence of the C_7_–C_9_ PFCA-decomposing bacterium *P. mosselii* 5(3) was determined.

Genomic DNA was isolated from a fresh culture biomass (a colony) of 5(3) strain grown on LB agar by the phenol-chloroform method according to the protocol (5). Sequencing was performed on the GenoLab M system (GeneMind Biosciences Co., Ltd., China), and the library was prepared with a ShotGun SG GM library preparation kit (cat. No SG_GM-96, Raissol, Russia). We obtained 2,154,440 paired-end reads of 150 bp in size. Quality control of reading was performed using “HTQC” (6). Low-quality (Q < 25), short (<100 bp) readings, and adapter sequences were removed using the Trimomatic v.0.39 software (7). Raw-filtered readings were *de novo* assembled using SPAdes version 3.15.4 software (8). Contigs shorter than 1,000 bp have been removed.

To identify the strain to species, we used the average nucleotide identity (ANI) value [https://www.ezbiocloud.net/tools/ani (9)] and digital DNA-DNA hybridization (DDH) [https://ggdc.dsmz.de/ggdc.php (10)] with default settings. The closest relative of the strain 5(3) is *P. mosselii* DSM17497 (T). The ANI value and DDH with the type strain *P. mosselii* DSM17497 (T) are 97.40% and 80.30%, respectively.

The strain 5(3) genome was annotated with the NCBI Prokaryotic Genome Annotation Pipeline version 4.6 (11). The assembly contained 5134 coding sequences, 4 rRNA clusters, and 65 tRNAs. The genome consists of 73 contigs with a total length of 5,676,241 bp. The N50 value is 165,927 bp, and the G-C content is 64.38%. The genome of *P. mosselii* 5(3) has haloalkane dehalogenase gene (dhaA) and haloacetate dehalogenase H-1 gene (dehH1), responsible for the degradation of fluorinated compounds.

## Data Availability

This genome project has been deposited at GenBank under the accession numbers JAUHUJ010000000 and BioSample number SAMN36271085, BioProject number PRJNA990579, and SRA accession number SRR25905155.
